# Improving working relationships with families in German early childhood interventions home visitors: a quasi-experimental training study

**DOI:** 10.1186/s40359-022-01009-x

**Published:** 2022-12-12

**Authors:** Anna K. Georg, Sophie Hauschild, Paul Schröder-Pfeifer, Lea A. Kasper, Svenja Taubner

**Affiliations:** 1 Institute for Psychosocial Prevention, University Hospital Heidelberg, Bergheimer Str. 54, 69115 Heidelberg, Germany; 2 Psychological Institute, University Heidelberg, Hauptstraße 47-51, 69117 Heidelberg, Germany

**Keywords:** Early childhood prevention/intervention, Continued professional development, Working relationship, Home visitations, Mentalizing, Healthcare professionals

## Abstract

**Background:**

Home visitation services within German Early Childhood Interventions (ECI) for families with a child aged 0–3 are mainly provided by frontline pediatric nurses and family midwifes. Home visitors are often challenged by difficult interactions with families. Mentalizing, the ability to understand mental states of oneself and others, is a key skill for building effective working relationships, which in turn positively affect intervention outcomes. The aim of this study was to investigate if a mentalizing skills training offered to home visitors active in German ECI contributes to continued professional development. We investigated, whether the training positively affected the quality of the working relationships with families as well as home visitors’ empathy, self-efficacy, and mentalizing.

**Methods:**

To test the effects of a single day mentalizing skills training on the working relationship in *N* = 73 ECI home visitors, we used a quasi-experimental design with repeated measures (T0, T1, T2, T3) across seven weeks in order to assess immediate change from baseline (T0) after the training (T2) and stability of changes at follow up (T3). A literature-based intervention was implemented before the training to estimate possible repeated measurement and expectational effects (T1). Primary outcome was the quality of the working relationship experienced by the home visitors. Secondary outcome criteria were empathy, work-related self-efficacy, self-reported and observer-rated mentalizing.

**Results:**

Significant positive change in the working relationship quality was observed at T2 and at T3. Results on the secondary outcomes were less consistent, with data indicating improvement in empathy and increase on some but not all components of mentalizing.

**Conclusions:**

This study provides preliminary evidence that brief mentalizing skills trainings may be an effective method for continuous professional qualification in frontline ECI home visitors who afterwards, experience better working relationships with families. Thus, training participation may positively impact efficacy and implementation of home visitations in ECI.

## Background

Early Childhood Interventions (ECI) are a composite of services for young children and their families provided when a child needs special support to ensure and enhance his/her personal development, strengthen the family’s own competences, and promote the social inclusion of the family and the child [1]. In Germany, ECI provide psychosocial support services to at-risk families to prevent adverse health outcomes or maltreatment, which affect an estimated 13% of young families [2] and about 300 000 children aged 0–3 years [3]. Services provided include assessment of family needs, parent counselling, and linking families with other resources in their communities such as health or social services. One intervention increasingly recognized for their effectiveness in preventing adverse outcomes and foster child health, and competent parenting, are home visitations [4, 5]. The home visiting services within the German ECI are mainly provided by healthcare professionals like pediatric nurses and family midwives, who have additional qualifications related to psychosocial care.

An effective working relationship between home visitors and parents has been regarded as a central mechanism in the implementation of successful home-based interventions targeting families [6, 7]. Home visitors with whom parents report having positive working relationships are experienced as honest and interested in the family’s needs [8]. However, home visitations place high demands on the professional’s skills needed to build an effective working relationship with the family [9, 10]. Forming a positive relationship seems to be particularly challenging if families are difficult to reach, e.g., if parents experience barriers to participate in ECI services, face problems to trust the support offered or if parents are ambivalent about change [11–13]. Professionals may find this constellation emotionally demanding and be confronted with feelings of helplessness, which in turn may hamper their engagement, reflection, and judgement [13]. In the context of difficult working relationships, home visitors’ perception of parents’ lack of cooperation, lack of responsiveness to outreach, or only partial compliance with interventions can impede their engagement and ability for empathic communication, reflection, and judgement, particularly in at-risk families [12, 14, 15].

Based on this literature, home visitors should be supported to develop skills that lead to effective and responsive working relationships, particularly with families at-risk and who are difficult to engage with [7, 9]. To this end, home visitors may benefit from being trained in skills for reflection and for maintaining an empathic attitude in the face of stressful parent-provider interactions. While implementation research in evidence-based home visiting ECI that aim at reducing child maltreatment demonstrated positive effects of home visitors’ training and supervision on program outcomes across medical, social, and mental health professionals [4], little research has been done on effective training practices that contribute to ongoing professional development in pediatric nurses and family midwives working in ECI [16, 17]. In addition, studies on the enhancement of reflective and emotional competencies in child welfare have mainly focused on students [18], leaving the question open as to what extent trainings are effective for frontline “on-the-job” home visitors.

Mentalizing has been studied as a core competency in mental health professionals for forming empathic relationships [19, 20]. It is defined as the ability to understand mental states of oneself or others that underlie overt behavior [21]. Mentalizing is typically operationalized as reflective functioning and is assessed with interviews on attachment relations (e.g., the Adult-Attachment-Interview [22]) or self-report-questionnaires. Another operationalization of mentalizing is mind-mindedness, which refers to the tendency to adopt an intentional stance in interactions with and in representations of others [23]. Mind-mindedness can be assessed by using speech samples and by counting mental descriptors parents use when describing their child [e.g., Adkins, Luyten and Fonagy [24]. Doing justice to the breadth of the concept, mentalizing has recently been described as an umbrella concept that overlaps with a range of related capacities, including aspects of social cognition, such as psychological mindedness or empathy [25]. Mentalizing means taking an empathic, inquisitive, non-judgmental and ‘not-knowing’ stance with respect to others’ mental states, and flexibly considering alternative perspectives. As such, it is a capacity regarded as particularly relevant to the helping relationship [20].

Mentalizing can be temporarily impaired when the emotional arousal is high, such as in the case of anger or anxiety [26] or when attachment related arousal is activated [27]. Thus, it is likely that high levels of emotional distress that can arise in challenging situations while working with families may limit home visitors’ capacity to mentalize. Such limitations can lead to interpersonal misunderstandings, disengagement, and cause ruptures in the working relationship [28]. Alternatively, mentalizing can reduce the likelihood of such ruptures and can contribute to ongoing professional development when such difficulties are successfully resolved. These experiences in turn support mentalizing and can lead professionals to experience greater self-efficacy and to engage with future clients more positively [29].

Mentalizing skills trainings for mental health professionals utilize guided reflective activities, self-experiential exercises, and mentalization-based therapy (MBT)-techniques to strengthen professionals’ capacity to mentalize in stressful situations. Two controlled studies investigated weekly mentalizing skills trainings for addiction counsellors [30] and psychology students [31] delivered over 20 weeks compared to psycho-educative trainings and found greater improvements in reflective capacity. Utilizing a two-day mentalizing skill training with adjunct case supervision in healthcare professionals, Welstead et al. [32] replicated the results of a previous pilot study by finding improvements of knowledge about MBT and improved attitudes about working with patients with personality disorders [33]. Similarly, Williams et al. [34] reported improvements after a two-day mentalizing skills training with adjunct supervision in knowledge about mentalization, empathy, metacognition, and confidence in working with personality disorders. Most of the improvements remained stable during a five-months follow-up with add-on supervisions. These findings suggest that mentalizing trainings can help novice and expert mental health professionals to improve mentalizing and together offer preliminary evidence for the positive effects of brief (2-days) trainings for this population.

Adult learning research suggests better the outcomes (i.e., knowledge, skills) the more actively involved learners are in evaluating their improvement and in reflecting on his or her experience in learning [35]. The meta-analysis demonstrated that particularly for skill acquisition, evaluating and reflecting on the targeted knowledge or practice is most effectful [35]. Based on this result, a mentalizing skills training, that actively engages home visitors in the learning process, could effectively improve the skills required to build effective and responsive working relationships.

The aim of this study was to investigate the research question if a brief one-day mentalizing skills training for a group of ECI home visitors positively effects their perception of the working relationship as well as their level of empathy, work-related self-efficacy, and mentalizing. Based on the initial evidence on brief mentalizing skills trainings [32, 34], the goal of this study was to investigate the effects of a one-day training that would fit into the already established training structure for home visitors within the German ECI.

Based on meta-analytic evidence on adult learning research, according to which active learner involvement leads to the best outcomes [35], we expected that the simple introduction of didactic content by reading a brochure about mentalizing would have little effect on our outcomes of interest. Building upon the literature on mentalizing skills training, we expected trainings’ effects beyond improved knowledge of mentalizing that are more specific to our trainings’ model. We hypothesized that the training would contribute to better working relationships on the part of home visitors with future clients (primary outcome) after the training (T2) and that the expected changes would still be evident four weeks later (T3). In addition, we explored effects on secondary outcomes, such as increased empathy and increased work-related self-efficacy at T2 and T3. Moreover, we expected improved mentalizing at T2 and T3. We did not expect to observe changes on any of the outcome parameter after implementing a literature-based intervention (T1).

## Methods

### Study design

We used a quasi-experimental study design with repeated measures whereby all eligible participants received a literature-based condition (brochure about mentalizing) and a one-day mentalizing skills training in a predefined order. The literature-based condition was implemented to estimate possible repeated measurement and expectational effects. The training took place at three different locations in Germany (Heidelberg, Cologne, and Berlin). Participants self-selected to the training location based on its proximity to their workplace.

After T0, all participants received the brochure and were asked to read it within a time frame of two weeks prior to the next assessment (T1) which was presented two days before the training. We expected that during a couple of working days participants would have the opportunity to implement new skills and experience a change of the working relationship. Therefore, T2 was presented to all participants seven days after the training. A follow-up assessment (T3) took place 28 days after the training. The time frame was chosen to specify the effects and to assess possible long-term effects found in previous brief mentalizing skills trainings [34].

Data collection was carried out online via SoSci Survey with an average of approximately 90 min completion time across participants and at each time point. The survey included self-report questionnaires and instructions for five-minute speech samples about difficult interpersonal situations at work at all time points. We also assessed satisfaction with both the training and the literature-based conditions. Audio files with participants’ speech samples were sent to the study center electronically after completing the online survey.

Participants were blind to the study’s hypotheses. It was impossible to blind trainers to experimental hypotheses. Raters of observer-based mentalizing were blind to time point.

The ethical approval for this study was obtained from Ethical Committee, Heidelberg University, Medical Faculty (No. S-309/2018 approved May 22, 2018). All participants gave their informed consent to participate in the study.

### Sample characteristics

Data was collected from January 2019 to March 2019. The study was coordinated by the Institute for Psychosocial Prevention, University Hospital Heidelberg. Participants were recruited from the nationwide network of the National Centre for Early Prevention (Nationales Zentrum Frühe Hilfen, NZFH), using websites, flyers, and Twitter and were asked to contact the study center when interested in study participation. We included professionals currently working as home visitors in ECI, including family midwifes or pediatric nurses, as well as lay volunteers. Certain ECI programs in Germany provide service by volunteers (e.g., within the service “Welcome” visits after birth) and 1.4% of young families receive visits by volunteers [2] which is why they were included in this study. Home visitors who usually reported typically seeing families only once without a follow-up were excluded from study participation. In a phone screening, inclusion and exclusion criteria were assessed and eligible home visitors were invited for study participation if they provided verbal and written informed consent.

### Study participants

**Fig. 1:**
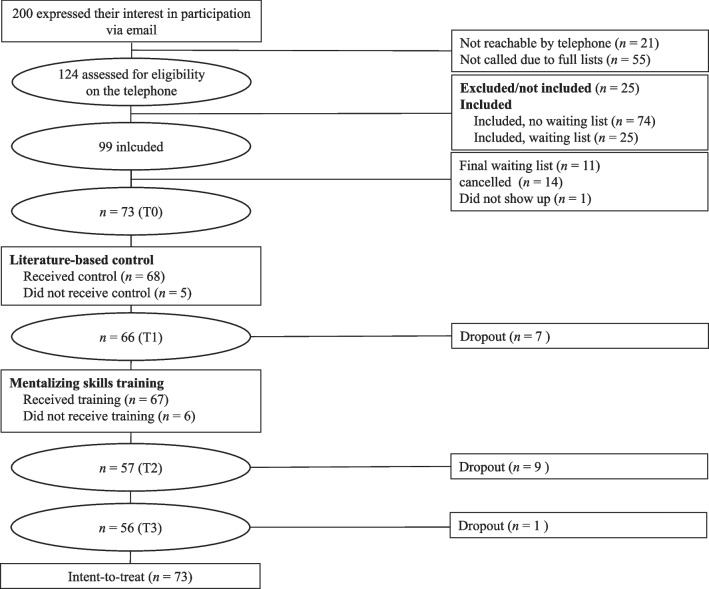
Participant flow chart. *Note.* T0 = pretest, T1 = assessment post literature-based intervention, T2 = assessment post training, T3 = follow-up assessment

Figure 1 displays the participant flow. Among the 200 interested parties, 124 were reached for a phone screen and assessed for eligibility. Twenty-five individuals were excluded from study participation; in most cases because they worked in administrative functions or did not work in the field of ECI. Of the 74 individuals that consented, *n* = 73 participated in T0. Twenty-five individuals were initially put on a waiting list, out of whom 14 were included after other participants cancelled. The remaining 11 individuals needed to be excluded due to training locations’ capacities that limited the number of participants.

Two (2.74%) participants were men and 94.5% were professionals in ECI trained as a midwife or a pediatric nurse. Two of the four volunteers were social workers. Mean age of participants was 50.82 (*SD* = 8.01; range 29 to 66). Participants reported an average of *M* = 16.71 years (*SD* = 9.14, range 2–31) of previous experience working in ECI and 66% reported having participated in a workshop on communication or working relationship in the past.

### Interventions

#### Mentalizing skills training

The manualized one-day (8-h) training in mentalizing skills for ECI home visitors was developed with Anthony Bateman based on the existing MBT skills training for mental health professionals [36] and the adaptive mentalization-based integrative treatment concept, which is a team-based approach to address the needs of complex clients [11]. The objective of the training was to improve the quality of future working relationships by strengthening mentalizing skills.

The training was delivered in a group setting and combined didactic teaching, guided reflections, experiential and simulation-based training, and video clips. The schedule was divided into eight sections focused on certain contents and tought competences, one sections to start off and close with, as well as several comfort breaks and a lunch break. The eight sections were: (1) Starting with an exercise: How do you know who I am?; (2) What is the meaning of mentalizing?; (3) Mentalizing stance and mentalizing communication; (4) Mentalizing problems; (5) Mentalizing of challenging working relationships in ECI; (6) Balancing one’s own mentalizing; (7) Strengthening parental mentalizing; and (8) Repair ruptures in working relationships. The last section included an overall reflection on the acquired skills and giving feedback.

Video clips on interpersonally difficult situations were shown to engage participants in mentalizing and demonstrate core theoretical concepts. Core theoretical concepts (e.g., defining mentalizing and stress-related mentalizing problems) and related skills were taught and practiced in group exercises and real- and role plays in small groups, including how to engage in self- and other-focused reflections, maintain and communicate a mentalizing stance in stressful situations, and how to repair ruptures in the relationship. Additionally, specific MBT techniques (e.g., stop, stand, and rewind) and supplementary tools (e.g., checklist for mentalization-based work involving for example questions on the practitioners perception of the family during the last visit in black/white or multifaceted) were taught and practiced in role plays. Home visitors’ experiences with all real- and role plays, and their perceived learning outcome were afterwards discussed within the larger group. To maximize the effect on interactions within the home visiting context, participants were encouraged to individual reflections on their working relationships and the discussion of personally relevant situations that affected the working relationship with families in the group.

The training was conducted by two female trainers who were trained in MBT (AKG and SH). Throughout the training, the trainers modelled a mentalizing stance in discussions about home visitors’ personal experiences and by pointing out moments where they struggled to do so, both in their communications with participants and with each other. Fidelity was ensured by following a training manual [37] that provided specific guidelines for each of the trainings’ sections as well as supporting training materials. In addition, the training was video recorded and the implemented training elements were checked against the training manual by the two trainers. All elements were implemented according to the training manual.

#### Literature-based intervention

The 10-page brochure covered the same core theoretical concepts in the day-long training along with illustrative examples and case descriptions relevant to ECI. One item included in T1 (‘knowledge about mentalization’) was used as a measure of whether the brochure was read as an estimate of fidelity.

### Measures

#### Working alliance inventory (WAI)

The WAI assesses the quality of a therapeutic relationship [38]. It has robust reliability [39] and good validity [40]. The WAI is comprised of 16 items scored on a 5-point Likert scale (*rarely* to *always*); higher scores signify a better working alliance with the client, according to the practitioner. The item wording was adapted to fit the context of ECI. Cronbach’s α was 0.91.

#### Therapists’ work involvement scales (TWIS)

The TWIS [41, 42] assess the quality of therapeutic engagement in the working relationship utilizing two scales: healing involvement (TWIS heal) and stressful involvement (TWIS stress). The scales obtained good validity and satisfactory reliability [41]. The TWIS contains 52 items which score on a 6-point Likert scale (*none/not at all/never* to *many/very/very often*) or a 4-point Likert scale (*never* to *very often*). We adopted the wording to the context of ECI. Cronbach’s α was 0.80 (TWIS heal) and 0.83 (TWIS stress).

#### Interpersonal reactivity index (IRI)

Two scales of the IRI [43] were used to assess empathy: change of perspective (cognitive dimension of empathy) and personal distress in emotionally difficult situations (emotional dimension of empathy) (4 items each, scoring on a 5-point Likert scale ranging from *does not describe me well* to *describes me very well*). The IRI has very good reliability and validity [43]. Change of perspective had an α of 0.84 and personal distress had an α of 0.79.

#### Generalized self-efficacy scale (GSES)

The GSES [44] reliably and validly assesses perceived self-efficacy with 10 items scored on a 4-point Likert scale (*not true* to *completely true*) with higher scores indicating more self-efficacy. Cronbach’s α was 0.83.

We utilized a range of measures for the different components of mentalizing due to the complexity of the construct [25] as well as the lack of previous training studies in this specific population. Self-report measures assessed attributional complexity, metacognition, and reflective functioning. The five-minute speech sample was coded using an observer-based measure to assess mind-mindedness while discussing a difficult interpersonal situation.

#### Attributional complexity scale-short (ACS)

Interest in mentalization was assessed with the ACS [45]. The questionnaire has been validated and contains seven items. In this study, it scored on a 5-point Likert scale ranging from *completely wrong* (1) to *completely correct* (5). Higher scores signify higher interest in mentalizing. Cronbach’s α was 0.81.

#### Metacognition self-assessment scale (MSAS)

The MSAS’ mastery scale assesses metacognition in problem solving (i.e., strategies which individuals use to exploit their knowledge of themselves and of others to solve psychological and interpersonal problems) and has sufficient validity and reliability [46]. The scale consists of five items which score on a 5-point Likert scale (*almost neve*r to *almost always*) with higher scores signifying more metacognition. This study is the first to use MSAS in a German sample; thus, it was translated via back-and-forth translation (English to German). The German MSAS had not been validated before. A Cronbach’s α of 0.71 in this study points towards a satisfying internal consistency.

#### Reflective functioning questionnaire (RFQ-8)

The RFQ-8 assesses quality of mentalizing with eight items on two scales, certainty (RFQ-c) and uncertainty of mental states (RFQ-u) [47]. Despite its low to satisfactory reliability and validity [47, 48], the RFQ-8 is the gold standard of self-reported mentalizing capacity. The items score on a 7-point Likert scale (*completely disagree* to *completely agree*). High or low scores on both scales indicate low mentalizing ability. Cronbach’s α was 0.84 (RFQ-c) and 0.58 (RFQ-u). Thus, RFQ-u was excluded from data analysis.

#### Five-minutes speech samples-mind-mindedness (FMSS-MM)

FMSS-MM [49] measures observer-rated mind-mindedness (MM) in speech samples. Five-minutes speech samples have been successfully used to assess quality/degree of mentalizing in parents’ narratives [24] using the MM coding manual [23]. FMSS-MM applies an adapted version of the MM coding manual to five-minute speech samples of home visitors’ narratives about difficult interpersonal situations. Two five-minute monologues per participant were collected within two tasks. The first task consisted of the presentation of a vignette which describes a typical difficult interpersonal situation in ECI (task 1). The second task prompts the participant to discuss a personally difficult interpersonal situation encountered while working as home visitors (task 2). After reading (task 1) respective after having described the personally difficult interpersonal situation (task 2), participants were asked to speak at least 5 min, answering four questions that pull for mentalizing (e.g., “How do you understand the family?”).

The coding of the speech samples counted the number of mind-minded words in the transcript using three scales: MM-other is the relative frequency (to the total words of the transcript) of mind-minded words related to another person (e.g., the mother was maybe *afraid* that I would take her child away); while MM-self is the relative frequency of mind-minded words related to the self (e.g., I *felt* a huge responsibility in this situation). MM-nk (MM not-knowing) is the relative frequency (to the total of mind-minded words) of words that reflect a not-knowing stance related to mind-minded comments (e.g., the mother was *maybe* afraid that I would take her child away). Higher values in MM-other and MM-self indicate more MM and higher values in MM-nk indicate a greater not-knowing stance. In sum, six scores were used for analyses: MM-other, MM-self, and MM-nk for task 1 (vignette) and task 2 (personally relevant situation).

Three raters (two authors of the study (AKG, SH) and an undergraduate psychologist) recalibrated the method by independent ratings of pilot data until reliability was deemed sufficient (*ICC* ≥ 0.82). To ensure blinding, transcripts were re-coded by a research assistant. FMSS-MM ratings were completed at the end of the study. *ICC*s were calculated for 50 of the 229 transcripts (21.83%) and ranged from MM-nk (0.72) to MM-self (0.87).

#### Knowledge about mentalization

A test was constructed to assesses knowledge about mentalization (e.g., demonstration of understanding the concept of mentalization) by utilizing 13 items in multiple choice format. Correctly answered items were summed into a sum score. At T1, the correctly answered question “knowledge about the basic theory of mentalization” was used to indicate whether the brochure had been read. The test had an α of 0.54 and thus it was excluded from further analysis.

#### Satisfaction with brochure and training

Satisfaction with the brochure and the training was assessed via two items, “general satisfaction” and “learning growths”, from the German Client Satisfaction Questionnaire (CSQ-8; [50]). The items scored on a 4-point Likert scale where higher scores indicate more satisfaction respective learning growths.

### Statistical analysis

A priori power analysis for the primary outcome was conducted assuming an average small to medium effect of f = 0.2 based on previous studies showing effects on attitudes working with challenging clients [32, 34]. An alpha of α = 0.05 and a power of β = 0.85 resulted in a sample size of *N* = 63 considering four measurement points and an assumed *ICC* of 0.2. Because of the exploratory nature of the study with regards to secondary outcomes, type I errors were not controlled for [51]. We corrected for multiple tests on the primary outcome scales (WAI, TWIS heal, TWIS stress) utilizing Bonferroni-Holm procedure.

To answer the question of whether or not the training had an effect on home visitor mentalizing and related outcomes, several multilevel models (MLM) were conducted with primary and secondary outcomes (level 1) nested within participants (level 2). A nesting of participants within training centers (level 3) was considered but dropped from the final model for lack of variance. Every model consisted of categorical time (T0, T1, T2 and T3) at level 1 as the main predictor, as well as age, additional training experience (dummy coded as 0 = none, 1 = some), and job experience in years as covariates on level 2, and a random effect for person to account for the nested data structure. Q-Q plots and sphericity plots were utilized to examine if the assumptions of MLM were met and no significant deviations were found.

Since the assessments were conducted online, forced-choice format was used to achieve data without missing values. The data was scanned for multivariate outliers utilizing Mahalanobis distance and no significant outliers were found.

Regression analyses with each outcome variable utilized as a criterion predicted by the rest of the outcome variables that were used as predictors were done to estimate multivariate collinearity between outcome measures [52]. Drop-out analyses comparing participants who dropped out during the study with completers regarding sample characteristics and outcome measures were performed with Mann–Whitney-U and *t*-Tests.

All statistical analyses were performed with RStudio version 1.2.5033 [53] using R version 3.6.3 [54].

## Results

### Assessment and intervention completion

Of the T0-sample, 76.71% took part in T3, while *N* = 17 dropped out (Fig. 1). Participants who dropped out did not significantly differ from completers regarding sample characteristics or outcome measures at baseline (*p*s < 0.053). The control question about the brochure was answered correctly by 93.15% of the participants. Thus, its implementation was regarded as largely completed. Of the 91.78% of participants who took part in the training, 22 took part in Heidelberg and Cologne sites and 23 in Berlin.

### Intervention outcomes

Table 1 displays baseline (T0) descriptive statistics and Pearson’s correlations of all outcome measures. Significant bivariate correlations were all in the expected directions and of small to moderate magnitude. The highest correlation was found between TWIS heal and IRI perspective (*r* = 0.61, *p* < 0.001). We found no evidence for multivariate collinearity between outcome variables (largest *R*^2^ = 0.48).

**Table 1:** Descriptive statistics and correlations of outcome variables at T0 of *n* = 73 home visitors in ECI

Variables	*M* / % (SD)	*SE*	*Mdn*	Range (min–max)	Skew-ness	*r* (*p*)													
						TWIS heal	TWIS stress	IRI distress	IRI perspec-tive	GSES	ACS	RFQ-8	MSAS	MM-other_v	MM-self_v	MMnk_v	MM-other_e	MM-self_e	MMnk_e
WAI	3.8 (0.43)	0.05	3.81	2 (2.69–4.69)	− 0.29	**.46 (< .001)**	− **.44 (< .001)**	− **.30 (.010)**	**.47 (< .001)**	**.42 (< .001)**	**.375 (.001)**	**.33 (.005)**	**.36 (.002)**	− .17 (.165)	− .02 (.844)	− .13 (.291)	− .08 (.519)	.00 (.984)	− .08 (.500)
TWIS heal	11.38 (1.02)	0.12	11.24	5.88 (8.72–14.6)	0.36		− .15 (.219)	− **.24 (.038)**	**.61 (< .001)**	**.33 (.004)**	**43 (< .001)**	**.29 (.013)**	**.41 (< .001)**	− .03 (.799)	− .04 (.734)	− .02 (.874)	− .05 (.686)	− .02 (.881)	− .12 (.317)
TWIS stress	3.87 (1.28)	0.15	3.77	7.36 (1.05–8.41)	0.71			− **.48 (< .001)**	− .18 (.118)	− **.30 (.010)**	.01 (.919)	− **.48 (< .001)**	− **.23 (.046)**	.07 (.556)	.02 (.857)	.07 (.567)	.03 (.807)	.12 (.310)	− .09 (.443)
IRI distress	8.85 (2.39)	0.28	9	13 (4–17)	0.71				− .20 (.093)	− **.41 (< .001)**	.07 (.531)	− **.34 (.003)**	− .21 (.078)	− .02 (.863)	− .01 (.941)	.20 (.099)	.20 (.089)	.07 (.593)	.02 (.872)
IRI perspective	16.48 (1.99)	0.23	17	8 (12–20)	− 0.20					**.38 (< .001)**	**.40 (< .001)**	**.32 (.006)**	**.37 (.002)**	− .21 (.081)	− .09 (.480)	− .12 (.314)	− .07 (.544)	− .03 (.828)	− .14 (.252)
GSES	30.34 (3.07)	0.36	30	17 (22–39)	0.15						.05 (.656)	**.26 (.030)**	**.27 (.023)**	− .09 (.473)	− .03 (.793)	.08 (.510)	− .06 (.640)	.04 (.752)	− .006 (.964)
ACS	4.19 (0.51)	0.06	4.14	1.86 (3.14–5)	− 0.22							.11 (.376)	**.38 (001)**	− .02 (.853)	.04 (.763)	− .05 (.702)	.06 (.639)	− .00 (.987)	.02 (.867)
RFQ-8	1.55 (0.74)	0.09	1.67	3 (0–3)	− 0.23								**.44 (< .001)**	.06 (.623)	.15 (.202)	− .11 (.365)	− .08 (.492)	− .06 (.600)	.01 (.925)
MSAS	19.52 (2.47)	0.29	19	11 (14–25)	0.33									− .00 (.978)	.21 (.072)	− .14 (.234)	.01 (.922)	.03 (.787)	.09 (.457)
MM-other_v	2.23 (1.35)	0.16	2.04	6.65 (0.36–7)	1.1										**.32 (.006)**	.19 (.103)	.18 (.131)	.14 (.253)	**.24 (.043)**
MM-self_v	0.96 (0.65)	0.08	0.84	2.67 (0–2.67)	0.65											− .18 (.124)	.09 (.443)	**.33 (.006)**	.00 (.997)
MMnk_v	33.87 (19.34)	2.28	31.11	92.86 (0–92.86)	0.6												**.44 (< .001)**	.08 (.488)	.22 (.069)
MM-other_e	1.51 (0.85)	0.1	1.32	4.11 (0–4.11)	0.82													.14 (.256)	**.26 (.030)**
MM-self_e	0.95 (0.65)	0.08	0.84	3 (0–3)	1.08														− .17 (.176)
MMnk_e	19.47 (15.81)	1.9	18	66.69 (0–66.69)	0.84														

The bolded numbers indicate significant (*p* < .05, *p* < .01, *p* < .001) correlations. *ACS* Attributional Complexity Scale, *e* refers to the ratings of the personal experience; *GSES* General Self-efficacy Scale, *IRI distress* personal distress subscale of the Interpersonal Reactivity Index, *IRI perspective* change of perspective subscale of the Interpersonal Reactivity Index, *MM-other* Mind-Mindedness other, *MM-self* Mind-Mindedness self, *MMnk* Mind-Mindedness not knowing, *MSAS* mastery subscale of the Metacognition Self-Assessment Scale, *TWIS heal* healing involvement subscale of the Therapists’ Work Involvement Scales, *TWIS stress* stressful involvement subscale of the Therapists’ Work Involvement Scales, *RFQ-8* certainty subscale of the Reflective Functioning Questionnaire; *v* refers to the ratings of the vignettes, *WAI* working alliance inventory

#### Effects on the working relationship (primary outcome)

There was a significant positive change in the working alliance (WAI) at T2 (β = 0.13, *p* = 0.012) but not at T3 (β = 0.07, *p* = 0.105). As expected, significant immediate and follow-up changes were observed in TWIS heal (T2: β = 0.32, *p* = 0.015; T3: β = 0.28, *p* = 0.024) and TWIS stress (T2: β = − 0.28, *p* = 0.038; T3: β = − 0.34, *p* = 0.020) indicating increased healing involvement and decreased stressful involvement pertaining to working with families immediately after the training and at follow-up. Table 2 shows the fixed effects of the multi-level models on the primary outcome.

**Table 2:** Fixed effects of the multilevel models on the working relationship (primary outcome), interpersonal distress, and general self-efficacy in *n* = 73 home visitors in ECI

	WAI		TWIS heal		TWIS stress		IRI distress		IRI perspective		GSES	
	Estimate [95% CI]	*p*	Estimate [95% CI]	*p*	Estimate [95% CI]	*p*	Estimate [95% CI]	*p*	Estimate [95% CI]	*p*	Estimate [95% CI]	*p*
*Predictors *(Intercept)	3.54 [2.96; 4.11]	**< .001**	12.04 [10.59; 13.49]	**< .001**	4.65 [2.66; 6.65]	**< .001**	12.60 [9.39; 15.81]	** < .001**	17.42 [14.59; 20.25]	** < .001**	26.82 [22.63; 31.01]	**< .001**
T1	− 0.05 [− 0.19; 0.09]	.485	− 0.00 [− 0.37; 0.37]	.996	− 0.03 [− 0.46; 0.40]	.878	0.30 [− 0.53; 1.14]	.479	− 0.30 [− 1.06; 0.47]	.447	0.11 [− 1.04; 1.25]	.855
T2	0.13 [0.05; 0.21]	**.012†**	0.32 [0.11; 0.53]	**.015†**	− 0.28 [− 0.52; − 0.05]	**.038†**	− 0.57 [− 1.05; − 0.08]	**.022**	0.10 [− 0.35; 0.54]	.666	0.07 [− 0.60; 0.74]	.840
T3	0.07 [− 0.01; 0.15]	.105†	0.28 [0.07; 0.50]	**.024†**	− 0.34 [− 0.58; − 0.10]	**.020†**	− 0.76 [− 1.25; − 0.27]	**.002**	0.29 [− 0.16; 0.73]	.210	0.56 [− 0.11; 1.24]	.103
*Covariates*												
Additional training	− 0.00 [− 0.16; 0.16]	.995	− 0.04 [− 0.45; 0.38]	.863	− 0.30 [− 0.84; 0.24]	.272	− 0.62 [− 1.54; 0.30]	.188	− 0.07 [− 0.89; 0.75]	.863	− 0.14 [− 1.36; 1.08]	.822
Age	0.00 [− 0.01; 0.02]	.441	− 0.01 [− 0.04; 0.01]	.354	− 0.01 [− 0.05; 0.03]	.564	− 0.06 [− 0.12; 0.00]	.054	− 0.02 [− 0.07; 0.04]	.518	0.08 [− 0.00; 0.16]	.059
Working experience	0.00 [− 0.00; 0.01]	.509	0.00 [− 0.01; 0.02]	.734	0.00 [− 0.02; 0.02]	.991	− 0.01 [− 0.05; 0.03]	.520	0.00 [− 0.03; 0.04]	.922	− 0.02 [− 0.08; 0.03]	.396

^†^*p*-value corrected for multiple testing utilizing Bonferroni-Holm. Reference level for all time contrasts (T1, T2, T3) is T0. *N* = 73

The bolded numbers indicate significant effects (*p* < .05, *p* < .01, *p* < .001). Reference level for all time contrasts (T1, T2, T3) is T0. *GSES* Generalized Self-efficacy Scale, *IRI distress *personal distress subscale of the Interpersonal Reactivity Index, *IRI perspective* change of perspective subscale of the Interpersonal Reactivity Index, *TWIS heal *healing involvement subscale of the Therapists’ Work Involvement Scales, *TWIS stress *subscale stressful involvement of the Therapists’ Work Involvement Scales, *WAI* Working Alliance Inventory

#### Effects on secondary outcomes

Significant immediate and follow-up changes were observed in the IRI distress (T2: β = − 0.57, *p* = 0.022; T3: β = − 0.76, *p* = 0.002), signifying decreased interpersonal distress experiences. Contrary to expectation we found no change in the IRI change of perspective scale and in the GSES. A significant change was observed in the ACS at T3 (β = 0.11, *p* = 0.046), demonstrating increased metalizing interest, whereas a change at T2 was not observed (Table 3). Similarly, a significant change demonstrating higher MSAS problem solving at T3 was observed (β = 0.84, *p* = 0.003), whereas we found no change in either time point on the RFQ certainty scale (Table 3).

**Table 3:** Fixed effects of the multilevel models on secondary outcome criteria in *n* = 73 home visitors in ECI

	ACS		MSAS		RFQ-8		MM-other_v		MM-self_v		MM-nk_v		MM-other_e		MM-self_e		MM-nk_e	
	Estimate [95% CI]	*p*	Estimate [95% CI]	*p*	Estimate [95% CI]	*p*	Estimate [95% CI]	*p*	Estimate [95% CI]	*p*	Estimate [95% CI]	*p*	Estimate [95% CI]	*p*	Estimate [95% CI]	*p*	Estimate [95% CI]	*p*
*Predictors * (Intercept)	4.55 [3.82; 5.28]		18.53 [15.48; 21.58]		0.49 [− 0.52; 1.51]	.341	14.86 [8.23; 21.48]		5.81 [2.45; 9.18]		56.56 [33.16; 79.96]		11.08 [6.34; 15.82]		6.66 [3.20; 10.13]		30.95 [12.25; 49.65]	
T1	− 0.05 [− 0.23; 0.13]	.599	− 0.23 [− 1.14; 0.68]	.616	− 0.10 [− 0.35; 0.14]	.409	− 1.63 [− 3.16; − 0.10]	**.037**	0.34 [− 0.47; 1.14]	.411	− 2.86 [− 9.52; 3.79]	.399	− 0.69 [− 1.96; 0.59]	.294	0.05 [− 0.84; 0.95]	.906	0.79 [− 4.92; 6.51]	.785
T2	− 0.01 [− 0.12; 0.09]	.811	0.49 [− 0.06; 1.04]	.080	0.12 [− 0.02; 0.26]	.083	2.64 [1.05; 4.24]	**.001**	− 0.73 [− 1.56; 0.11]	.087	7.16 [0.31; 14.00]	**.041**	− 1.17 [− 2.50; 0.17]	.086	− 0.85 [− 1.78; 0.09]	.075	4.24 [− 1.70; 10.17]	.162
T3	0.11 [0.00; 0.21]	**.046**	0.84 [0.28; 1.39]	**.003**	0.13 [− 0.01; 0.26]	.078	− 0.85 [− 2.46; 0.76]	.301	0.73 [− 0.10; 1.57]	.085	11.19 [4.29; 18.08]	**.001**	− 0.59 [− 1.97; 0.80]	.407	− 0.19 [− 1.16; 0.78]	.696	− 0.11 [− 6.28; 6.06]	.972
Additional training	− 0.04 [− 0.24; 0.17]	.733	− 0.33 [− 1.24; 0.57]	.473	0.28 [− 0.01; 0.56]	.058	− 1.20 [− 3.29; 0.90]	.262	0.38 [− 0.68; 1.45]	.477	− 1.01 [− 8.36; 6.33]	.787	0.03 [− 1.46; 1.52]	.970	0.36 [− 0.73; 1.46]	.515	2.13 [− 3.75; 8.00]	.478
Age	− 0.01 [− 0.02; 0.01]	.383	0.02 [− 0.04; 0.08]	.462	0.02 [− 0.00; 0.04]	.094	− 0.06 [− 0.19; 0.06]	.334	− 0.02 [− 0.08; 0.05]	.586	− 0.43 [− 0.87; 0.01]	.058	− 0.06 [− 0.15; 0.03]	.196	− 0.03 [− 0.09; 0.04]	.424	− 0.19 [− 0.54; 0.17]	.295
Working experience	− 0.00 [− 0.01; 0.01]	.838	0.00 [− 0.04; 0.05]	.823	0.00 [− 0.01; 0.01]	.855	− 0.07 [− 0.18; 0.04]	.191	− 0.06 [− 0.11;− 0.00]	**.035**	− 0.02 [− 0.40; 0.35]	.902	− 0.02 [− 0.10; 0.05]	.579	− 0.04 [− 0.10; 0.01]	.131	− 0.22 [− 0.52; 0.08]	.147

The bolded numbers indicate significant effects (*p* < .05, *p* < .01, *p* < .001). Reference level for all time contrasts (T1, T2, T3) is T0. *ACS* Attribution Complexity Scale, *e* refers to the ratings of the personal experiences, *MM-other* Mind-Mindedness other, *MM-self* Mind-Mindedness self, *MM-nk* Mind-Mindedness not-knowing stance, *MSAS* mastery subscale of the Metacognition Self-Assessment Scale, *RFQ-8* subscale certainty of the Reflective Functioning Questionnaire, *v* refers to the ratings of the vignettes. For the intercept *p* < .001 for all variables except for RFQ-8 *p* = .341

The assessment of observer-rated MM yielded mixed results with significant positive immediate (T2: β = 7.16, *p* = 0.041) and follow-up changes (T3: β = 11.19, *p* = 0.001) observed for MM-nk, as rated on the vignettes. There was a significant decrease in MM-other at T1 (β = − 1.63, *p* = 0.037) and a significant increase of MM-other at T2 (β = 2.64, *p* = 0.001). There were no significant changes in MM-self and MM measures rated on personally difficult situations (Table 3).

#### Satisfaction

Table 4 describes the satisfaction with brochure and training. Overall, participants were satisfied with both interventions. More participants deemed their learning growth to be significant after the training compared to after reading the brochure.

**Table 4:** Subjective ratings of satisfaction and learning growth

	Brochure *n* (%)	Training *n* (%)
Satisfaction		
Highly satisfied	15 (23.8)	16 (28.1)
Generally satisfied	36 (57.1)	29 (50.9)
Marginally satisfied	11 (17.5)	12 (21.1)
Dissatisfied	1 (1.6)	0
Learning growth		
Marked learning growth	18 (28.6)	23 (40.4)
Was a little helpful	40 (63.5)	27 (47.4)
No learning growth	5 (7.9)	7 (12.3)
Made things more difficult	0	0

*n* = 66 for the brochure. *n* = 57 for the training

## Discussion

This study investigated the immediate and follow-up effects of a one-day-mentalizing skills training that aimed to improve the working relationship with families in ECI frontline home visitors by using a quasi-experimental within-subject design with repeated measures. Previous controlled and uncontrolled studies on mentalizing skills trainings for mental health professionals demonstrated effects on mentalizing and related capacities [30, 31, 34], knowledge about mentalizing [34], attitudes of working with patients [33], and confidence [34] immediately after the training and in a follow-up after a brief 2-day training [34]. With this study we added to this research by investigating if a brief training for ECI home visitors that focused on improving mentalizing skills for a better parent-provider relationship, exerts positive effects on the working relationship and additional secondary outcomes.

Our sample consisted mainly of family midwifes or pediatric nurses with considerable experience as home visitors in ECI. As hypothesized, we observed immediate and follow-up changes in all working relationship indicators, except for the working alliance, where the change disappeared at follow-up. Thus, our results showed that home visitors experienced a better working alliance, increased healing involvement, and decreased stressful involvement in their relationships with families following the training. Results on the explorative secondary outcomes were more inconsistent. There was a decrease in interpersonal distress, but no change in perspective taking or work-related self-efficacy. Some, but not all, self-reported and observer-rated mentalizing measures showed improvements. Most of the participants were satisfied with the brochure and training. In the following we discuss the results in detail.

The overall goal of the training to increase the quality of the working relationships experienced by ECI home visitors was reached. In particular, we found that the perceived working alliance with families was better after the training. The self-experiential parts of the training that encouraged participants to reflect on individual cases using the mentalizing skill tools provided may have resulted in a short-term effect on the working relationship as experienced by the home visitor. In addition, home visitors’ engagement within the working relationship improved; specifically, constructive engagement, (TWIS heal), as defined by relational skills and agency, positive in-session feelings, and a constructive handling of difficult situations, increased. At the same time, unconstructive engagement (TWIS stress), which is characterized by frequent difficulties, avoidant coping, and negative feelings while working with families, decreased. These results add to previous studies on brief mentalizing stills trainings positively affecting the attitude and confidence of working with challenging clients [32, 34]. Thus, our results suggest that training mentalizing skills may improve home visitors’ capacity to build effective working relationships probably in part by increasing their confidence for resolving challenging situations constructively.

Based on robust results demonstrating the significance of the working alliance for positive outcomes in psychotherapy [55] and the relevance of a positive working relationship in the ECI-context [7], it is possible that an increased quality of the working relationship also contributes to better outcomes when working with families. The lack of a follow-up effect on the working alliance, however, could suggest that more in-depth practice or further supervision is needed for the skills learned in the one-day training to sustain positive effects on the working alliance over time. This is consistent with suggestions that mentalization supervision be provided to health care professionals for sustained effects of the skills training [32]. Further support for this notion comes from the beneficial effects of reflective supervision on home visitations’ outcomes [4].

Regarding secondary outcomes, the reduction of personal distress (IRI distress) is in line with the change of the working relationship, demonstrating a general effect in terms of fewer feelings of helplessness and anxiety in emotionally difficult interpersonal situations related to home visitations. Less emotional reactivity in these situations may positively affect empathic reactions to and behaviour toward the families. Contrary to expectations and to previous evidence using another empathy measures [34], change of perspective (IRI perspective), the cognitive aspect of empathy, did not increase with the training. This is especially surprising as the scale is strongly related to TWIS heal, where the expected improvement was found. It is possible that the training, which focused on mentalizing within the working relationship, does not provide sufficient training to have an effect on the more general reflective and empathic abilities assessed with these scales.

Similarly, we did not observe the expected change in self-efficacy at work after the training. This is contrary to the finding of [34], reporting an improvement in confidence of working with patients with a personality disorder which is related to the concept of self-efficacy. Here as well, the training’s focus on promoting mental processes related to specific problems, particularly in the working relationship, might have contributed to the lack of changes observed on the GSES, which captures behavior-based solutions to work-related challenges in general. Mental processes within the relationship are better reflected in the MSAS or TWIS where we found a change following the training. In the future, assessments should assess the confidence in using mentalizing skills related to challenging interpersonal situations in order to assess change more specific to the training’s model.

An overall effect on mentalizing measures was not observed which is surprising giving previous evidence about training effects on reflective capacities [30, 31, 34]. Instead, the results were inconsistent, with two measures showing no change after the training or at follow-up (RFQ certainty, MM-self). Our results suggest that the training increases two specific components of mentalizing: interest in mentalizing (ACS) and the capacity to approach problems via mental processes (MSAS). Home visitors’ capacity to mentalize family members (MM-other) and to take on a mentalizing stance (MM-nk), even in challenging situations as measured with the FMSS, increased. It is possible that through practicing maintaining an inquisitive, not-knowing stance while reflecting on typically challenging situations with families, participants’ capacity to mentalize others during difficult interactions increased.

The inconsistent results on the mentalizing measures may be attributable to the short training duration. The one-day training, compared to previous longer trainings [30, 31, 34] probably gives too little time to practice and for change to consolidate. Increasing the training duration, adding ongoing supervision, or providing more tools for deliberate practice of reflective capacities outside of the training could result in greater mentalizing improvements [35, 56]. The lack of association between changes on self- and observer-rated mentalizing, while consistent with the general lack of correspondence between self-reported and observer-rated measures [57], points to a need to validate the FMSS-MM measure for the assessment of mentalizing.

While the training and the brochure were perceived positively by most of the participants, future research might focus on the small subsample that did subjectively not benefit from the training and determine what if any adaptations to the training can be implemented to better fit these individual’s needs. Also, the results of the satisfaction questionnaire reflect that a brochure seems to already contribute to a subjective growth among some participants. This was however, not reflected in our outcome measures and requires future investigations.

### Limitations

First of all, due to the quasi-experimental study design and the lack of an independent control group, we cannot exclude that the changes observed from T0 to T2 and to T3 were caused by other factors than the training. Other environmental or individual factors, as well as repeated measurement or expectation effects might have contributed to the changes. The latter possibility was aimed to be addressed with the literature intervention. Except for the decrease in MM-other, there were no changes observed from T0 to T1. We could have detected both repeated measurement effects or changes due to participants’ expectations about having received an intervention in general. In addition, the general lack of effects on some variables (e.g., GSES) and the distinct patterns of results even within self-report measures, contradict the notion of a bias due to a general repeated measurement effect. Thus, we have some reason to believe that repeated measurement or expectation effects did not cause the observed changes although we cannot completely rule out their influence. Additionally, improvements in skills are less likely after simply introducing new learning content [35] and thus we expected that the brochure only minorly contributed to the effects on most of our outcome criteria. The decrease in MM-other may even indicate a detrimental effect on reflective qualities from receiving only theoretical knowledge about mentalizing [31].

However, we cannot exclude, and it is indeed possible, that reading the brochure exerts an additional effect on the outcomes measured after the training. In fact, the meta-analysis on learning methods demonstrated that the combination of different learning methods will more likely have optimal positive benefits [35]. Although the training itself already combined multiple methods, the introduction of knowledge prior to the training could additionally engage learners and positively affect subsequent learning processes. This could still be the case although our results did not show significant changes after reading the brochure (T1). Thus, facilitators who want to ensure training effects based on our results, should provide participants with the brochure in advance. Future studies may utilize a randomized-controlled design with an independent control group in order to exclude combination effects. Another limitation is that the significant effects on the secondary outcomes need to be interpreted with caution due to the exploratory nature of their investigation. Future research is needed to replicate the results in confirmatory studies and in addition ensure fidelity regarding the trainers’ metalizing stance by an external rater.

As expected, effects on outcome measures were small. It is possible that a longer training would contribute to stronger effects [35]. In addition, training effects may have been larger in a fully trained sample. Although the intervention completion rate in both conditions was higher than 90%, there were also some non-attenders in the analyzed intent-to-treat sample. In addition, we predominantly used self-report measures, making the study more prone to expectation effects. This limitation is offset to some degree by the use of the observer-rated mentalizing measure, despite its need for further validation. Future studies may also investigate the impact on actual day-to-day interactions with families and include parents’ perceptions of changes in the working relationship.

This study may have a selection bias towards home visitors who were possibly vigilant to challenges in the working relationship, as the majority had participated in trainings targeting interpersonal competencies in the past. Due to low participation rates of volunteers, this group needs replication in future studies.

## Conclusion

This study provided preliminary evidence that a one-day mentalizing skills training that draws on experiential and simulation-based methods and is preceded by a didactic brochure is a feasible and effective method for continuous professional development in frontline health professionals in the field of ECI. The training positively affected the perceived working relationship with families, possibly by teaching mentalizing skills that help home visitors to effectively resolve common challenges in the work. Health professionals in ECI who want to improve their working relationships with families in their day-to-day work, will likely benefit from participating in the training. Within better working relationships with families, efficacy and implementation of home visitations in ECI may increase. Including more MBT-tools to support application of the skills in practice, more self-experiential elements in the training, or supplemental supervision, may further increase and consolidate training effects. Future studies may adapt the training accordingly and experiment with different training lengths.

Together with the existing evidence of brief mentalizing trainings’ effects, our study provides the first evidence for their application in early prevention health professionals and their potential to effectively contribute to a perceived better working relationship with the clients they serve. The results of our study should encourage more rigorous research through controlled studies and by involving outcomes that reflect the perspective of the families.

## Data Availability

The datasets used and analyzed during the current study are not publicly available due their containing information that could compromise the privacy of research participants but are available from the corresponding author on reasonable request.

## Abbreviations

ACS: Attributional Complexity Scale-Short
CSQ-8: Client Satisfaction Questionnaire
ECI: Early childhood intervention
FMSS-MM: Five-Minutes Speech Samples Mind-Mindfulness
GSES: Generalized Self-efficacy Scale
ICC: Interclass correlation
IRI: Interpersonal Reactivity Index
MBT: Mentalization based treatment
MLM: Multilevel models
MM: Mind-mindfulness
MM-nk: Mind-mindfulness not-knowing
MSAS: Metacognition Self-Assessment Scale
NZFH: Nationales Zentrum Frühe Hilfen
RFQ-8: Reflective Functioning Questionnaire
TWIS: Therapists’ Work Involvement Scales
WAI: Working Alliance Inventory
